# Trends in psychological distress among adolescents in Ireland: survey data from 2018 to 2023 & lived experience perspective

**DOI:** 10.1007/s00127-025-03026-8

**Published:** 2026-01-29

**Authors:** Niamh Dooley, Bel Aghedo, Sophie Mae Harrington, Amber Johnson, Jack Kirby, Naoise Owens, Isobel Solan, Georgia-May Staunton, David McEvoy, Mary Cannon, Louise Arseneault

**Affiliations:** 1 Social, Genetic and Developmental Psychiatry Centre, King’s College London, London, UK; 2 Department of Psychiatry, Royal College of Surgeons in Ireland University of Medicine and Health Sciences, Dublin, Ireland; 3 Independent researcher, Galway, Ireland; 4 Independent researcher, Worcester, UK; 5 Independent researcher, Dublin, Ireland; 6 FutureNeuro Research Ireland Centre, Royal College of Surgeons in Ireland University of Medicine and Health Sciences, Dublin, Ireland

**Keywords:** Adolescents, Epidemiology, Mental health, Lived experience

## Abstract

**Purpose:**

This study aimed to estimate the prevalence of poor mental health, repetitive self-harm, and suicide attempts among adolescents in Ireland between 2018 and 2023; to identify demographic groups at elevated risk; and to assess changes in these outcomes across the time period.

**Methods:**

The Planet Youth survey is a repeated cross-sectional study in which 21,340 secondary school students aged 15–19 years and 588 early school leavers were surveyed across seven local authorities in Ireland. Participants anonymously self-reported mental health status, lifetime repetitive self-harm, and suicide attempts. We used multilevel logistic regression, accounting for school and regional clustering, to estimate population prevalence rates, identify risk groups, and assess temporal trends. Young people with lived experience of adolescent mental health problems provided their insights on survey methodology and findings.

**Results:**

Averaged across the 5-year period, 19.6% reported poor mental health, 12.1% repetitive self-harm, and 8.4% a lifetime suicide attempt. Transgender/gender-diverse adolescents and early school leavers were most likely to report these outcomes. By 2022/23, rates of poor mental health and suicide attempt had returned to pre-COVID levels, however, rates of repetitive self-harm continued to rise. Lived experience reflections highlighted the contributions of service inaccessibility and the role of cultural trends.

**Conclusion:**

Findings highlight high levels of psychological distress among Irish youth. There was significant heterogeneity of risk across subgroups, with implications for both universal and targeted interventions. Continued surveillance of adolescent mental health and the expansion of accessible youth mental health services remain key priorities.

**Supplementary Information:**

The online version contains supplementary material available at 10.1007/s00127-025-03026-8.

## Introduction

International evidence suggests that adolescent mental health has deteriorated over the past two decades [1, 2]. Compared to the adolescents of the 2000 s and 2010 s, adolescents of the 2020 s tend to report more symptoms of depression and anxiety, and have a higher probability of being diagnosed with a mental illness [1]. Emotional distress levels appear to have begun rising between 2010 and 2015, with another rise observed during the COVID-19 pandemic [3–7]. Reasons for these trends are unclear, but theories include a changing understanding and language around mental health, the content and ubiquity of digital media, increased loneliness, and greater economic inequality (for reviews see [1, 5, 8]).

Changes in adolescent mental health are not just observable from self-report surveys. Data from hospital emergency departments and population-based surveys shows rising proportions of self-harming adolescents, particularly females [9–11]. The age of onset of self-harm appears to have decreased, with rising proportions of children aged 10–14 presenting to hospitals in the UK and Ireland with self-harm related injuries [10, 12]. Growing rates of self-injurious behaviour among children and adolescents constitutes a public health crisis, not only due to the profound emotional distress underlying these behaviours, but also because of the risk of suicide and the substantial burden placed on emergency department resources [13].

Adolescent mental health trends since COVID-19 remain unclear, with some, but not all, studies pointing to worsening trends. The Health Behaviours of School-aged Children surveys of 11–15-year-olds in over 40 countries showed that life satisfaction dropped significantly between 2018 and 2022, with the largest drop observed for female adolescents [14]. In England, the Mental Health of Children and Young People survey found that the proportion of adolescents with a probable mental disorder did not return to pre-pandemic levels, but have risen each year since 2021 [15]. Similarly, the Finnish School Health Promotion survey found that the proportion of adolescents (mean age 15.8) with clinical levels of generalised anxiety, depression, and social anxiety symptoms increased from pre-COVID to 2021 and remained at these higher levels in 2023 [16]. The Youth Risk Behavior Survey in the United States showed that the return to pre-COVID levels was measure dependent; persistent feelings of sadness or hopelessness have plateaued at around 40% two years after COVID (2021–2023), but rates of suicidal ideation and attempt returned to their pre-COVID ranges [17].

Anonymous surveys in the community are an invaluable source of information about the psychological wellbeing and self-harming behaviours of adolescents. Young people are more likely to disclose self-harm and suicidal phenomena via anonymous self-report surveys than in-person assessments [18, 19] and health service records can under-estimate the numbers of adolescents in the community struggling with their mental health. For instance, only half of adolescents with a probable mental disorder seek formal support from a healthcare professional [20, 21] and hospital records of self-harm and suicide attempts only capture the most severe injuries [22, 23].

Repeated cross-sectional surveys are invaluable sources of data to track mental health of adolescents, as a population group. This involves regularly surveying a defined population with the same measures used at each time point, so that we can establish change from one year to the next. School-based surveys with an opt-out consent process have high participation rates and are representative of school-going adolescents from a range of social and economic backgrounds. Repeated cross-sectional surveys allow us to focus on population trends (e.g., changes from the year 2018 to 2023) as opposed to age-related changes (e.g., changes from age 16 to 20). In this study, we use bi-annual cross-sectional surveys from secondary school students to estimate prevalence rates for this population.

Our aims were to: (1) estimate the national prevalence of self-ascribed poor mental health, repetitive self-harming, and suicide attempt in Ireland between 2018 and 2023; (2) identify demographic groups in the adolescent population at particular risk for these mental health problems; and (3) examine changes in the rates of these mental health problems, and whether they have returned to pre-COVID-19 pandemic levels. We also sought insights on the methods and results of this study from a group of young people in Ireland and the UK with lived experience of mental health problems in their adolescence. The purpose of this was to compare findings with young people’s real-life experiences, and to consider alternative interpretations of results that may differ from researcher assumptions.

## Method

### Study design & setting

**Fig. 1:**
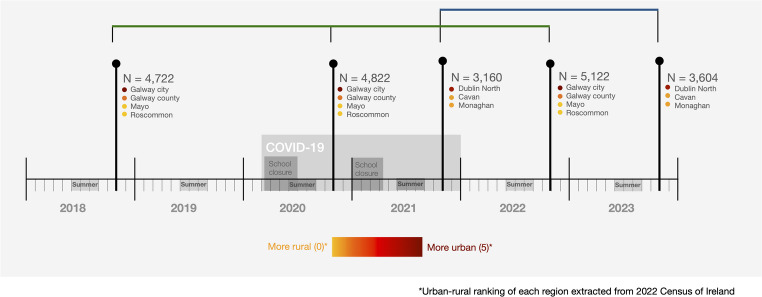
Details of surveys, including participating regions and timeline of data collection in context of COVID-19

The Planet Youth study is an international initiative based on the Icelandic Prevention Model [24]. The study aims to understand risk and protective factors for substance use and mental health problems among adolescents on a regional level. In Ireland, the study began in 2018 across four local authorities (Galway city, Galway rural, Mayo, Roscommon), extending to three more in 2021 (Dublin North, Cavan, Monaghan; Fig. S1). Surveys were repeated every 2 years, between October and December. Given different start years, not all counties collected data in same years. All data collected between 2018 and 2023 were used for this analysis (Fig. 1). Except in 2018, when it was delivered on paper, the survey was delivered digitally via tablets or computers and took approximately 40 min to complete, during school time. Teachers and survey coordinators delivered standardised instructions on administering the survey. See https://planetyouth.ie for more details.

### Participants

The target population for this study was secondary school students who had completed the Junior Certificate exams the previous academic year. This cohort is predominantly aged 15–16, with older students (17–19) participating in some schools. In the Dublin site, the target population included younger students (≤ 14 years) who were removed to ensure comparability with other regions. While this study focused on secondary school students, pupils in non-traditional educational centres known as “Youthreach centres” were also invited to participate. Individuals typically enrol in these centres either because they dropped out of school, are at risk of dropping out, or face barriers to traditional schooling. This group is referred to as “early school leavers”. Data from 15 Youthreach centres was collected, with at least one centre from each local authority. All institutions were invited to take part by local study leads. As per Planet Youth protocol, schools with less than 23 respondents were merged with a neighbouring school to protect anonymity.

### Target population representativeness

Each regional sample is largely representative of secondary school students who completed the Junior Certificate the previous year within that region. School participation rates ranged from 70 to 100% (mean = 97%), and the opt-out consent process guaranteed a high student participation rate within selected year groups (Table S3-4). The number of eligible versus participating students within each school was not recorded systematically across all years and regions. However, using publicly available education records, we estimated that, on average, 80% (SD = 6%) of students who sat the Junior Certificate exams the previous year participated in the survey. Compared to the 2022 Census of Ireland (aged 0–18 years), our sample had a similar break-down of ethnicities (e.g., 78% White Irish in this sample, compared to 82% in the Census) but included a higher proportion of young people from rural regions (Fig. S2).

### Consent & ethics

The study employed an opt-out consent process wherein participants were automatically included unless they, or their parents, declined participation. This approach is justified in contexts where participation poses minimal risk and explicit opting-in may reduce response or introduce selection bias. All eligible pupils and their parents received information detailing the study’s objectives, procedures, and privacy safeguards. After survey completion, participants were provided information on social care and wellbeing services in their community. Ethical approval for the data collection was granted by the Research Ethics Committee of the Royal College of Physicians in Ireland (RECSAF 82 and 144).

### Measures

#### Psychological distress measures

##### Poor mental health

Participants were asked *“How would you rate your mental health?”* and responded on a 5-point Likert scale. Responses were dichotomised into *“Bad”/“Very Bad”*, and “*Okay”/“Good”/“Very good”*, to focus on perceived problematic mental health.

##### Repetitive self-harm

Lifetime self-harm was captured by the question: *“During your lifetime*,* how often have you harmed yourself on purpose (e.g.*,* scratching*,* burning*,* preventing wounds from healing*,* punching)?”.* Response options included *“Never”*,* “Once”*,* “Twice”*,* “3–4 times”* and *“5 times or more”.* Those choosing “*5 times or more*” were considered repetitive self-harmers. We focus on repetitive self-harm to avoid including occasional and common behaviours such as nail-biting or skin picking. Furthermore, greater numbers of self-harm episodes are proportional to the risk of suicide in young people [13] and five or more self-harming events (per year) is a feature of the DSM-5 diagnosis of Non-Suicidal Self-Injury [25].

##### Suicidal attempt

Lifetime suicidal attempt was captured by the question “*Have you ever attempted suicide?”* (2021, 2023) or “*Have you ever made an attempt to complete suicide*” (2018, 2020, 2022) and participants answered “*yes*” or “*no*”. 

#### Demographic factors

Participants reported their gender (male, female, transgender/non-binary, prefer not to say). Those identifying as trans/non-binary, or who preferred not to say were merged into a trans/gender-diverse group. We opted to group those selecting “prefer not to say” with the trans/gender-diverse group, as non-disclosure may indicate gender questioning. Other research has shown those who choose “prefer not to say” are more similar to trans and gender-diverse young people compared to those identifying as male or female in terms of their mental health [26]. Urbanicity was a continuous variable ranging from 0 (most rural) to 5 (most urban), capturing the average urbanicity of the local authority. This 6-band categorisation was designed by the Central Statistics Office of Ireland (https://www.cso.ie/en/releasesandpublications/ep/p-urli/urbanandrurallifeinireland2019/introduction/). Maternal education level and family make-up were also recorded, with the latter determined by parents living with the respondent (two parents/one parent/one parent and their partner or a grandparent/other). “Other” family make-ups included living with grandparent(s) only, other relatives, living alone, in foster care, or with a host family.

There were some differences in demographic variables across survey years. For example, in 2018 only “male” and “female” were provided as gender options, and in the 2020 and 2022 surveys, “non-binary” and “prefer not to say” were included, but “transgender” was not (Table S1).

### Statistical analysis

We used logistic multilevel regression models throughout, each including two nested random effects (schools within local authorities), with fixed factors depending on the research question.

To estimate population prevalences of poor mental health, repetitive self-harm, and suicide attempt, we conducted three intercept-only multilevel models with the intercept approximating average prevalence over all available years, schools and regions. The intraclass correlation coefficient (ICC) was calculated using the latent variable approach for binary outcomes (ICC = τ^2^/(τ^2^ + π^2^/3), where τ^2^ is the between-cluster variance for each random effect, and π^2^/3 represents residual variance).

To identify high-risk groups, we added the following fixed factors: gender identity, age group, maternal education level, family make-up, urbanicity, and timing of survey (COVID-19 period or not). Based on existing research [17, 27–29], we hypothesised that female and trans/gender-diverse adolescents would report significantly higher levels of each mental health outcome compared to males. Separately, we investigated differences between early school leavers and secondary school students, controlling for all covariates above. We expected early school leavers to report higher levels of all mental health outcomes than those in mainstream schools [20].

To estimate whether the rates of each outcome has changed since the COVID-19 period (2020/21–2022/23), we included a two-level categorical time fixed factor in a multilevel model, in addition to gender, age, maternal education, and family make-up. To investigate whether rates of each outcome *returned* to pre-pandemic levels, we limited the analysis to a subset of regions which had collected surveys before, during, and after COVID-19 (2018, 2020, 2022). We included a three-level categorical time variable in addition to the demographic covariates mentioned above. We hypothesised that prevalence of each outcome would be significantly higher post-COVID compared to pre-COVID, based on findings from England [15].

The R code for this analysis is available on https://rpubs.com/dooleyr. A restricted version of the data can be downloaded from the Irish Social Science Data Archive (https://www.ucd.ie/issda).

### Lived experience input

A group of lived experience experts aged 17 to 26 with experience of adolescent mental health difficulties aided with the interpretation of the findings. These young people were recruited via mental health charities in Ireland (spunout) and the UK (McPin Foundation). A discussion of the methods, results, and the strengths/limitations of the study took place during two online focus groups, on 5th March (10 members) and 20th March (7 members) 2025. All quotes have been edited in collaboration with those quoted, and names were changed to protect the identities of members.

## Results

A total of 21,340 secondary school students aged 15–19 from 113 schools across Ireland completed the survey. There were similar numbers of males and females (49.5% vs 47.8%) and 2.2% (*n* = 480) were transgender or gender-diverse. The majority of participants were aged 15 (31.6%) and 16 years (58.9%; Table 1). See Table S5 for sample characteristics split by survey year.

### Population prevalence of mental health outcomes

**Fig. 2:**
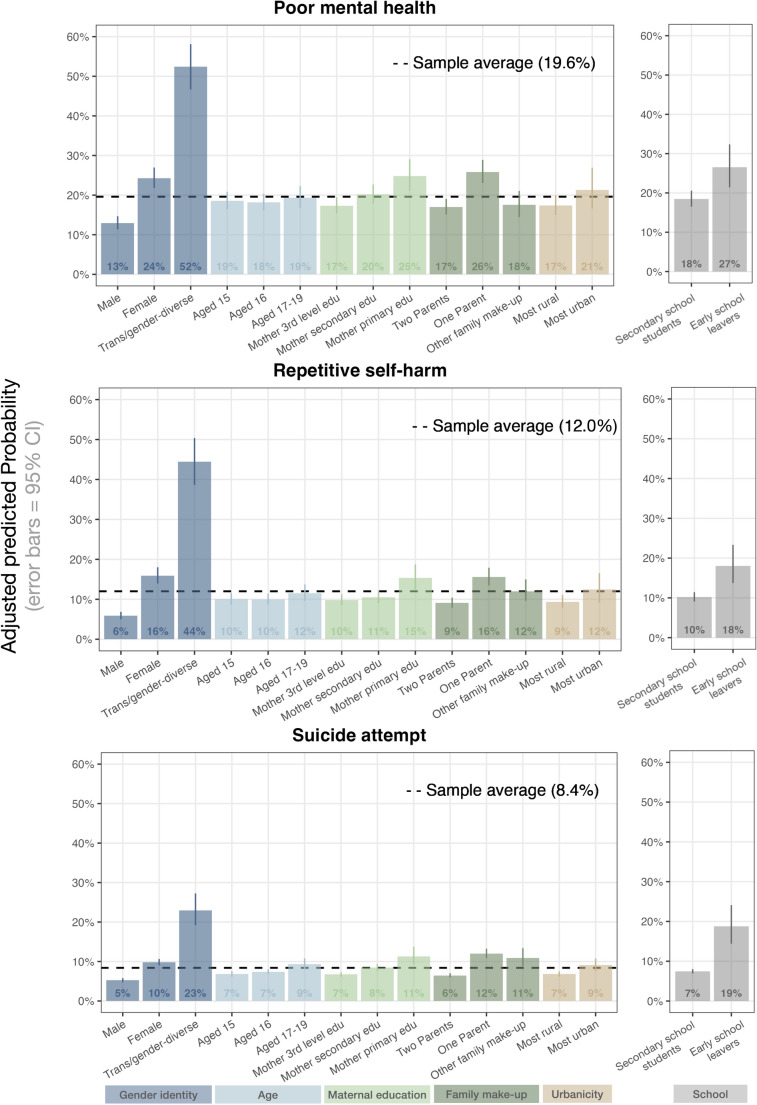
Adjusted prevalence estimates for sub-groups (bars) and average prevalence for the secondary school sample (hatched horizontal lines)

Approximately 1 in 5 secondary school students said their mental health was “bad” or “very bad” (19.6%; 95% CI = 11.8–30.4%; Fig. 2), 1 in 8 reported repetitively self-harming (12.1%; 95% CI = 5.4–19.5%) and 1 in 12 reported attempting suicide in their lifetime (8.4%; 95% CI = 4.7–13.0%). Confidence intervals were wider for poor mental health compared to repetitive self-harm and suicide attempt, reflecting lower precision and greater uncertainty in the population estimate. Variation across schools was greater than variation across local authorities, though these clustering variables accounted for < 5% of the variance combined (intra-class correlation coefficients were between 2% and 4% for school and ≤ 1% for local authority).

Among adolescents who reported poor mental health, 38% engaged in repetitive self-harm. Of those that engaged in repetitive self-harm, 41% also reported attempting suicide in their lifetime.

### Demographic risk-groups

The group with the highest adjusted probability of each outcome was trans/gender-diverse individuals. This group had an almost 13-fold increased odds of reporting repetitive self-harm, 7-fold increased odds of poor mental health, and 5-fold increased odds of lifetime suicide attempt, compared to males. Females were also significantly more likely than males to report all outcomes. Older adolescents (age 17+) were significantly more likely to report a suicide attempt and repetitive self-harm compared to 15-year-olds, but there were no other significant differences across age-groups. Lower maternal education levels (i.e., lower than 3rd level degree) were associated with higher odds of these adverse outcomes, as were one-parent families and other family make-ups, compared to two-parent families (Table 1).

A total of 588 early school-leavers also completed the survey. These individuals were significantly older than the secondary school participants, with 70.2% aged 17 or older (*χ*^2^ = 2282.5, *p* <.001). They were less likely to be living in a two-parent home (50.5% vs 78.3%; *χ*^2^ = 269.4, *p* <.001) and less likely to report mothers with college degrees (20.4% vs 58.9%; *χ*^*2*^ = 501.4, *p* <.001). Early school leavers were significantly more likely to report all mental health outcomes, even after adjustment for age and household socioeconomic factors (Table 1). Predicted probabilities of each outcome, after adjustment for covariates, were as follows: subjectively poor mental health (26.6%; Fig. 2), repetitive self-harm (18.1%), and suicide attempt (18.9%). Among early school-leavers who reported poor mental health (*n* = 176), 53% engaged in repetitive self-harm. Of those who engaged in repetitive self-harm, 72% also reported attempting suicide in their lifetime, which represents a greater overlap than in secondary school students.

**Table 1:** Differences in the odds of each outcome across demographic groups

	Group *N* (%)	Poor mental health		Repetitive self-harm		Suicide attempt	
		OR (95% CI)	*p*	OR (95% CI)	*p*	OR (95% CI)	*p*
**Secondary school students (** *N* ** = 21** **,430)**							
*Gender*							
Male"?>	10,617 (49.5%)	Reference					
Female	10,243 (47.8%)	**2.16 (2.00–2.33)**	**< 0.001**	**3.02 (2.73–3.35)**	**< 0.001**	**1.97 (1.76–2.20)**	**< 0.001**
Trans/gender-diverse	480 (2.2%)	**7.41 (6.08–9.03)**	**< 0.001**	**12.81 (10.38–15.80)**	**< 0.001**	**5.38 (4.25–6.82)**	**< 0.001**
*Age*							
15 years	6,777 (31.6%)	1.03 (0.95–1.11)	0.502	1.01 (0.91–1.11)	0.923	0.92 (0.82–1.03)	0.141
16 years	12,615 (58.9%)	Reference					
17–19 years	1,961 (9.2%)	1.08 (0.96–1.23)	0.203	**1.17 (1.01–1.37)**	**0.040**	**1.29 (1.09–1.52)**	**0.003**
*Family make-up*							
Two parents	16,782 (78.3%)	Reference					
Single parent	3,740 (17.5%)	**1.71 (1.56–1.86)**	**< 0.001**	**1.84 (1.66–2.04)**	**< 0.001**	**1.97 (1.75–2.21)**	**< 0.001**
Other family make-up	811 (3.8%)	1.04 (0.86–1.25)	0.711	**1.37 (1.10–1.70)**	**0.004**	**1.77 (1.40–2.23)**	**< 0.001**
*Mother education*							
Third level degree	12,626 (58.9%)	Reference					
Secondary school	4,966 (23.2%)	**1.21 (1.11–1.32)**	**< 0.001**	1.08 (0.97–1.20)	0.145	**1.28 (1.14–1.44)**	**< 0.001**
Primary school	802 (3.7%)	**1.58 (1.33–1.88)**	**< 0.001**	**1.66 (1.36–2.04)**	**< 0.001**	**1.77 (1.42–2.21)**	**< 0.001**
Not known	2,889 (13.5%)	1.08 (0.97–1.21)	0.146	0.97 (0.84–1.12)	0.681	1.16 (0.99–1.35)	0.060
Urbanicity (0–5)*		1.07 (0.97–1.19)	0.186	1.09 (0.98–1.22)	0.131	**1.09 (1.02–1.16)**	**0.007**
**All students (*N* = 22**,**018)**							
*School type*							
Secondary school	21,430 (97.3%)	Reference					
Centre for early school leavers	588 (2.7%)	**1.60 (1.23–2.07)**	**< 0.001**	**1.94 (1.41–2.65)**	**< 0.001**	**2.87 (2.07–3.97)**	**< 0.001**

Models also control for COVID-19 period (2020–2021). In **bold**: p-values < 0.05 (Bonferroni-adjusted p-threshold = 0.0167). * 0 = Most rural, 5 = Most urban

### Changes over time

For subjectively poor mental health, we observed a non-linear (inverted-V) effect of time, with rates peaking during the COVID-19 pandemic. Rates of poor mental health decreased in all regions after COVID (Fig. 2). An increase in repetitive self-harm during COVID occurred predominantly in urban regions (Dublin North; Galway City). The direction of change following the pandemic appeared region-dependent, with five local authorities showing an increase in the rates of self-harm, and two showing a decrease or plateau post-pandemic. As with self-harm, rates of suicidal attempt peaked during COVID to a much larger extent in urban regions (Dublin North; Galway City) than rural regions. Six of the seven regions showed a decline or plateau in rates of suicide attempt in the two years post-COVID, while one showed a small increase.

We found that the prevalence of poor mental health decreased from 22.0% during COVID to 16.2% two years later (OR = 0.68, 95% CI = 0.63–0.74, *p* <.001). The prevalence of suicide attempts also decreased albeit by a lesser magnitude from 8.1% to 7.1% (OR = 0.87, 95% CI = 0.77–0.97, *p* =.016). However, the proportion of adolescents reporting repetitive self-harm rose from 9.7% during COVID, to 11.1% two years post-COVID (OR = 1.16, 95% CI = 1.05–1.29, *p* =.004).

**Fig. 3:**
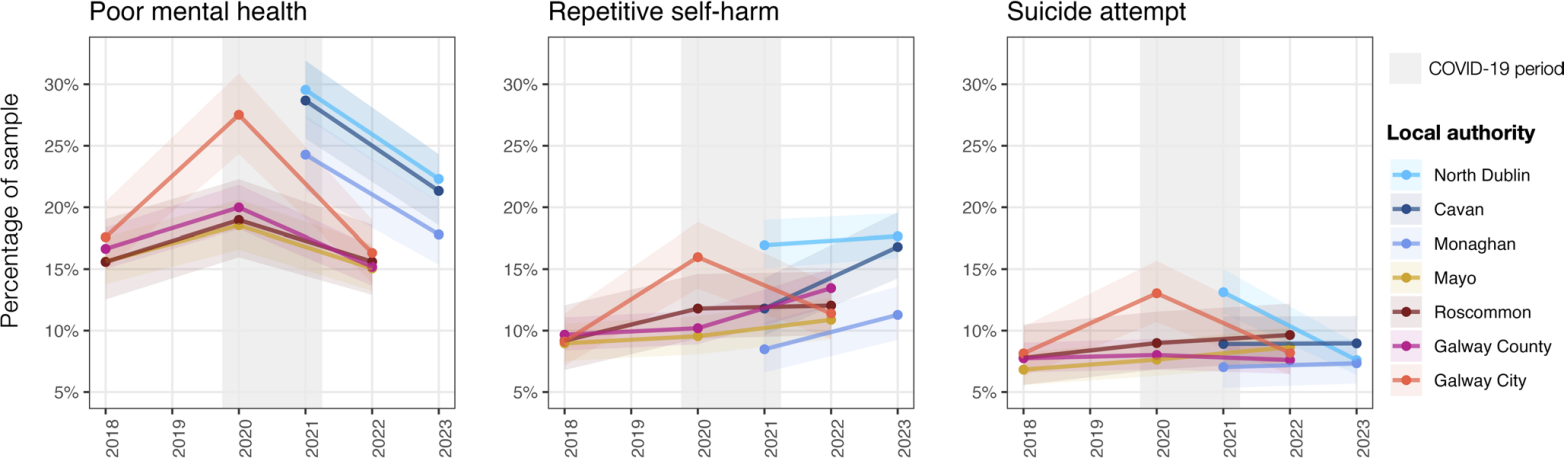
Changes in the unadjusted proportion of each outcome from 2018 to 2023, by local authority

Did rates of psychological distress return to pre-pandemic levels? This analysis was limited to regions with data before, during, and after COVID-19. Post-COVID rates of subjective poor mental health and suicide attempt returned to pre-COVID rates (i.e., no significant differences between pre- and post-COVID prevalence). However, rates of self-harm were significantly higher post-COVID (11.7%, 95% CI = 10.3–13.2%) compared to pre-COVID (9.0%, 95% CI = 7.8–10.3%). Our hypothesis of rising rates over the study period was therefore only supported for repetitive self-harm. As expected, higher odds of all mental health outcomes were observed during COVID-19 compared to pre-COVID.

Given that the proportions of trans/gender-diverse individuals changed over time (Table S5), and given they had particularly high rates of all outcomes, we re-ran the analyses excluding this group to ensure the observed trends were not an artefact of changing demographics. In a sample of young people identifying as male or female only, the same patterns were observed over time, and intraclass correlation coefficients pointed to the same levels of clustering within schools and local authorities (Fig. S5; Supplemental Results).

## Discussion

This study adds important findings from Ireland to the international discussion on how adolescent mental health has changed over the past five years. This novel data, reflecting the experiences of over 20,000 adolescents, shows that rates of subjectively poor mental health and suicide attempt have largely recovered from highs during the COVID-19 pandemic, but rates of repetitive self-harm continue to rise. This study comes at a time of significant societal concern about teenage mental health. The relatively high rates of suicide attempt across the full sample suggests the need for universal and early preventative interventions. However, our results also highlight significant heterogeneity among adolescents, and the demand for tailored interventions for more complex-needs groups like trans/gender-diverse youth and early school leavers.

### Trends over time: indicative of a teen mental health crisis?

Whether our findings support claims of a mental health crisis in Ireland depends somewhat on which baseline period we compare to, and what measure we focus on. For instance, we did not observe rapidly rising rates of all three measures of psychological distress within the 6-year study period (2018–2023). Only repetitive self-harm appeared to become more prevalent over time. However, the prevalence of suicide attempt in this sample *is* higher than previous estimates in secondary school students in Ireland; in 2018, a nationally-representative survey of secondary school students aged 12–19 in Ireland found a lifetime suicide attempt rate of 6%, which is 2.4% lower than our estimate, albeit with a wider age range [20]. Further back in 2009, a school-based survey of 1,096 adolescents aged 15–16 found that 5.2% had self-harmed five times or more in their lifetime [30], which is less than half the proportion observed in this study. While methodological differences exist between these studies, this provides tentative evidence for an increase in repetitive self-harm and suicide attempt rates between the period covered by this study (2018–2023) and the prior decade in Ireland, which aligns with international trends [1, 5].

The extent to which changes in self-reported distress convey a crisis also depends on how they translate to health service use. Questions about help-seeking were introduced in the 2023 survey, and indicated that of those reporting poor mental health, repetitive self-harm and a suicide attempt, just 39%, 47% and 55% accessed professional support for a mental health issue, respectively. This is in line with other research showing only about half of adolescents with probable mental illness receive professional support in Ireland and England [20, 21].

### Rising rates of repetitive self-harm

The proportion of repetitive self-harm in this general population sample rose by ~ 3% from 2018 to 2022. While this rise may seem small, it should not be dismissed for two reasons. First, repetitive self-harm is predictive of hospitalisation and suicidality and therefore has potentially devastating consequences for individuals and the health system [23]. Second, this rise triangulates with increasing proportions of adolescents presenting to primary and secondary clinics with self-harm injuries in Ireland [29, 31], which is echoed in the international literature [9, 32].

Why did we find a rise in repetitive self-harm but not in subjectively poor mental health or suicide attempt? The lived experience group suggested several possible reasons. First, long wait times for professional support or other barriers to access [33] may have left time for maladaptive coping strategies to emerge. Second, adolescents of the 2020 s report greater loneliness and perceive lower support from family and peers than previous generations [4, 34]. Finally, the visibility of self-harm in TV, film, and online may increase awareness, reduce stigma, and even glamorise self-harm. Greater visibility of self-harm and suicide on screens has been empirically shown to be associated with increased rates of self-harm in adolescents [35, 36].

School and local authorities captured just 5% of the variance in repetitive self-harm. Other studies have also found low levels of clustering of self-harm in schools [37, 38]. Although the absence of school- and area-level clustering points toward individual-level determinants of self-harm, rising rates across all regions, particularly among females, indicates that broader social influences should be explored such as media (Fig. S4) [9, 32, 38].

**Fig. 4:**
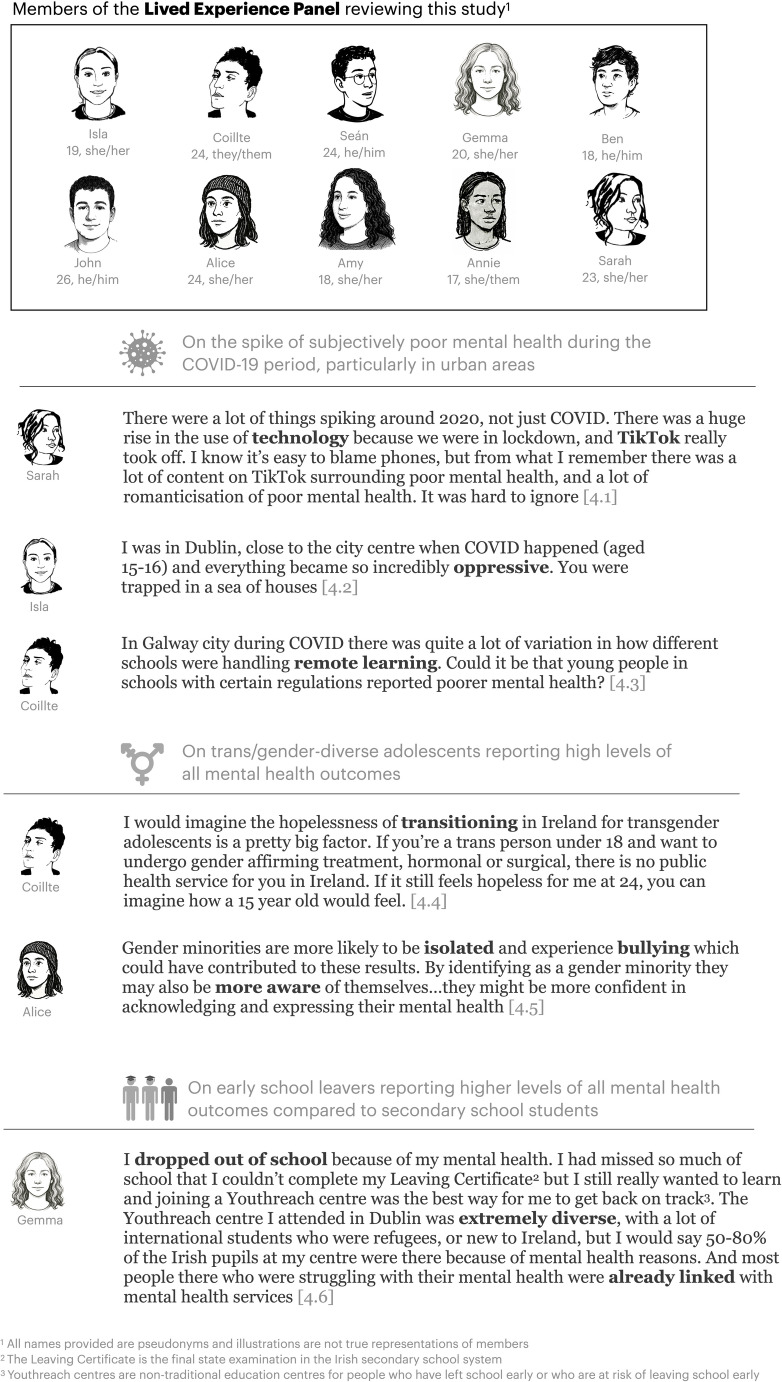
Lived experience reflections on findings

### Trans & gender-diverse youth: a small group with complex risks

The 480 adolescents identifying as trans or gender-diverse made up just 2.2% of the sample but had the highest risk of all mental health outcomes than any other demographic group by a considerable magnitude. Almost a quarter had made a suicide attempt and almost half had repetitively self-harmed. The lived experience panel was largely unsurprised by these findings, and the magnitude of group differences are comparable to other studies [28, 39–41]. There is a complex interplay of risk factors for trans and gender-diverse youth from dysphoric distress, to societal discrimination. One lived experience expert added that trans/gender-diverse youth may have a heightened self-awareness and therefore may be more likely to identify and report mental health problems (Fig. 4, quote 4.4). A trans/gender-diverse member of the lived experience group proposed that the poor mental health of this group is likely attributable, at least in part, to the lack of gender-affirming services in Ireland (Fig. 4, quote 4.5).

The concept of gender diversity has been hotly debated in societal and political spheres, often becoming a proxy issue for wider ideological conflicts [42]. However, the mental health of trans and gender-diverse young people is at risk of being overlooked amidst the discourse. Growing numbers of young people are identifying as trans/gender-diverse [43, 44] underscoring the need to develop care pathways tailored to their needs. Community-integrated interventions or online networks may be best suited to support wellbeing of this small but vulnerable group. A recent review showed that more high-quality clinical trials are needed to establish which interventions are effective at reducing suicide risk in this group [45].

### Risk of suicide among early school-leavers

The largest difference between early school-leavers and their school-going peers was in suicide attempt rates, with early school-leavers at almost threefold increased odds of reporting an attempt, even after controlling for potential confounds. A bi-directional relationship between early school departure and poor mental health is well supported [46, 47]. Indeed, a lived experience member who dropped out of school mentioned that a large proportion of people in her training centre had left school due to mental health difficulties. However, this member also noted a range of reasons including recent migration and specific educational needs, highlighting the diversity of this group.

Further research should aim to understand the modifiable predictors of poor mental health and self-injurious behaviours in this diverse group. While the lived experience perspective suggests that many early school leavers are already connected with mental health services (Fig. 4, quote 4.6), interventions could include suicide prevention supports at the institutional level and greater integration between mental health services and these educational centres.

### Post-COVID recovery

A key part of future pandemic preparedness will be to evaluate the long-term effects of COVID-19 and related lockdowns. We already know that the mental health of young people was disproportionally affected [48, 49]. Our study adds that adolescents from urban areas reported poorer outcomes during COVID, compared to those from more rural areas. For instance, in urban areas rates of suicide attempt in 2020/21 were almost double that of non-COVID years (Fig. 3). This pattern was previously found by two small cross-sectional studies of COVID-19 in Asia [50, 51], and may have important implications for the allocation of resources to urban youth in future emergencies.

Our findings provide some cause for optimism: that rates of poor mental health and suicide attempt have returned to their pre-COVID levels. However, there are caveats to this optimism. First, many individuals experienced a long-lasting deterioration of mental health during COVID which this repeated cross-sectional data cannot capture. Second, other studies in Ireland have found significant declines in life satisfaction in adolescents of the same age between 2018 and 2022, demonstrating the measure-specificity of findings [52]. And third, the post-COVID data remains limited and future phases of this survey will be needed to support inferences of recovery. 

### Limitations

First, this sample was not designed to be representative of the Irish adolescent population. That said, there is very high coverage of schools and students within participating local authorities, extending to non-mainstream education centres (Tables S3-4). Second, adolescents from urban areas are under-represented in this sample, but the numbers surveyed in cities or suburbs remained substantial (*n* = 5,770) and our sampling bias is in the opposite direction to the majority of psychological research. Third, the wording of questions or response options varied slightly across years, which may have led to biased estimates (Table S1). For instance, in 2018 only two gender options were provided (male and female), meaning that the overall proportion of trans/gender-diverse youth in this sample (~ 2%) is likely to be an underestimation. Fourth, there is inherent subjectivity in our measures. For example, the lived experience panel noted that the question *“how would you rate your mental health”* is subject to interpretation of the time range in question and conceptualisation of mental health. Fifth, not all demographic risk groups (e.g., race/ethnicity, sexual orientation, neurodiversity) could not be investigated due to data limitations. Finally, while this repeated cross-sectional data lends itself to analysis of trends, we cannot exclude the possibility that unmeasured time-varying confounders account for changes over time.

## Conclusions

Research from several other countries suggests increasing levels of self-reported mental health problems among adolescents over the past decade. This paper adds the Irish perspective to the evidence base, and advances our knowledge of adolescent mental health post-COVID. Encouragingly, our results suggest a return to pre-COVID levels for subjective reports of poor mental health and suicide attempts. Our data also points to the need for universal interventions to combat high population-wide problems and targeted interventions for specific at-risk groups.

## Supplementary Information

Below is the link to the electronic supplementary material.

Supplementary Material 1

## Data Availability

Some of the data used in this study is available via application to the Irish Social Science Data Archive (ISSDA), https://www.ucd.ie/issda/accessdata/issdadatasets/. Specifically, data from 4 local authorities (Galway county, Galway city, Mayo, Roscommon) is currently live on this archive, while data from the remaining 3 local authorities (Dublin, Monaghan, Cavan) will be deposited in Winter 2025/6.
