# Supplementary material for: Trends in psychological distress among adolescents in Ireland: survey data from 2018 to 2023 & lived experience perspective
Source: Soc Psychiatry Psychiatr Epidemiol. 2026 Jan 29;61(7):1279–91. doi: 10.1007/s00127-025-03026-8 (PMC13424716; doi:10.1007/s00127-025-03026-8)
Supplement: Supplementary file 1 — Supplementary Material 1 [file 127_2025_3026_MOESM1_ESM.docx]

# Supplementary Material

## Supplementary Tables

**Table S1.** Survey item wording and original response levels

|  | **Regions:** Galway, Mayo, Roscommon | | | **Regions:** North Dublin, Cavan, Monaghan | |
| --- | --- | --- | --- | --- | --- |
|  | **2018** | **2020** | **2022** | **2021** | **2023** |
| **Gender** | Are you male or female? | How would you describe your gender? | How would you describe your gender? | How would you describe your gender? | How would you describe your gender? |
|  | * Male  * Female | * Male  * Female  * Non-binary  * Prefer not to say | * Male  * Female  * Non-binary  * Prefer not to say | * Male  * Female  * Non-binary and/or trans  * Prefer not to say | * Male  * Female  * Trans-gender  * Non-binary  * Prefer not to say |
| **Maternal education** | What is the highest level of schooling your mother completed? (If you are mostly brought up by a foster mother you answer for her) | What is the highest level of education your mother/carer completed? | What is the highest level of education your mother/carer completed? | What is the highest level of education your mother / carer completed? | What is the highest level of education your mother / carer completed? |
|  | * Graduated from a university  * Started university but didn’t finish  * Graduated from technical college or apprenticeship  * Started technical college or apprenticeship but didn’t finish  * Graduated from secondary school  * Started secondary school but didn’t finished  * Primary school or less  * I don’t know/doesn’t apply | * Postgraduate Degree (Masters or Doctorate)  * Degree from University  * Diploma from a technical institute  * Completed Leaving Certificate  * Completed Junior Certificate  * Completed Primary School or less  * I don’t know / doesn’t apply | * College or University  * Secondary School  * Primary School  * Don’t know | * College or University Course  * Secondary School  * Primary School  * Don’t know | *College or University Course  *Secondary School  *Primary School  *Don’t know |
| **Family make-up** | I live with…  (Choose only ONE option, the one that suits best) | I live with…  (Choose only ONE option, the one that suits best) | Who do you live with? (Choose the option that suits best) | I live with… (choose the one that suits the best) | I live with… (choose the one that suits the best) |
|  | *I live with my mother and father equally  *Mother but not father  *Father but not mother  *Mother and her partner  *Father and his partner  *Grandparent/s and mother/father  *Only Grandparent/s and not mother /father  *I live with friends  *I live on my own  *I live in a different arrangement (foster family, guardian etc.) | * I live with both my parents  *Mother but not father  *Father but not mother  *Mother and her partner  *Father and his partner  *Grandparent(s) and mother/father  *Only grandparent(s) and not mother/father  *I live in a different arrangement (foster family, carer, other relatives, etc.)  *I am an exchange student and live with a host family | * I live with both my parents  * Mother but not father  * Father but not mother  * Mother and her partner  * Father and his partner  * Grandparent(s) and mother/father  * Only grandparent(s) and not mother/father  * I live in a different arrangement (foster family, carer, other relatives, etc.)  * I am an exchange student and live with a host family | * I live with both my parents  * One parent  * A parent and another adult(s)  * Other adults (i.e. extended family, foster family etc.) | * I live with both my parents  * One Parent  * A Parent and another adult(s)  * Other adults (i.e. extended family, foster family etc.)  * A host family |
| **Subjective mental health** | How would you rate your mental health? | How would you rate your mental health? | How would you rate your mental health? | How would you rate your mental health? | How would you rate your mental health? |
|  | * Very good  * Good  * Moderate  * Bad  * Very bad | * Very good  * Good  * Okay  * Bad  * Very bad | * Very good  * Good  * Okay  * Bad  * Very bad | * Very good  * Good  * Okay  * Bad  * Very bad | * Very good  * Good  * Okay  * Bad  * Very bad |
| **Lifetime self-harm** | During your lifetime have you harmed yourself on purpose? (such as scratching, cutting,  burning, preventing wounds from healing, punching) | During your lifetime have you harmed yourself on purpose?  (e.g., scratching, burning, preventing wounds from healing, punching) | During your lifetime have you harmed yourself on purpose? (e g , scratching, burning, preventing wounds from healing, punchin) | During your lifetime have you harmed yourself on purpose? (e.g., scratching, burning, preventing wounds from healing, punching) | During your lifetime have you harmed yourself on purpose? (e.g., cutting, scratching, burning, preventing wounds from healing, punching) |
|  | * Never  * Once in lifetime * Twice in lifetime * 2-4 times in lifetime  * 5 times or more often | * Never  * Once  * Twice  * 3-4 times  * 5 times or more often | * Never  * Once  * Twice  * 3-4 times  * 5 times or more often | * Never  * Once  * Twice  * 3-4 times  * 5 times or more often | * Never  * Once  * Twice  * 3-4 times  * 5 times or more often |
| **Lifetime suicide attempt** | Have you ever made an attempt to complete suicide? | Have you ever made an attempt to complete suicide? | Have you ever made an attempt to complete suicide? | Have you ever attempted suicide? | Have you ever attempted suicide? |
|  | * Yes  * No | * Yes  * No | * Yes  * No | * Yes  * No | * Yes  * No |

**Table S2.** Descriptive statistics for the secondary school sample (N=21,430)

|  | **N (% of total)** |
| --- | --- |
| **Gender identity (N = 21,340)** |  |
| Male | 10617 (49.5%) |
| Female | 10243 (47.8%) |
| Trans & gender diverse | 480 (2.2%) |
| **Age group (N = 21,353)** |  |
| 15 | 6777 (31.6%) |
| 16 | 12615 (58.9%) |
| 17-19 | 1961 (9.2%) |
| **Ethnicity (N = 16,621)** |  |
| White Irish | 12903 (77.6%) |
| White non-Irish | 1937 (11.7%) |
| Black, Asian or other minority ethnicity | 1781 (10.7%) |
| **School grade (N = 21,094)** |  |
| 4th Year (Transition Year) | 15444 (72.1%) |
| 5th Year | 5352 (25.0%) |
| LCA/QQI | 221 (1.0%) |
| 6th Year | 77 (0.4%) |
| **Family make-up (N = 21,333)** |  |
| 2 Parents | 16782 (78.3%) |
| 1 Parent | 2760 (12.9%) |
| 1 Parent +other | 980 (4.6%) |
| Other | 811 (3.8%) |
| **Maternal education level (N = 21,283)** |  |
| College or University | 12626 (58.9%) |
| Secondary School | 4966 (23.2%) |
| Primary School | 802 (3.7%) |
| Don’t know/Doesn’t apply | 2889 (13.5%) |
| **Region (N = 21,430)** |  |
| Dublin North | 3368 (15.7%) |
| Cavan | 1656 (7.7%) |
| Monaghan | 1740 (8.1%) |
| Mayo | 4414 (20.6%) |
| Roscommon | 1812 (8.5%) |
| Galway County | 6038 (28.2%) |
| Galway City | 2402 (11.2%) |

**Table S3.** Proportion of secondary schools in each region that participated (as a percentage of all registered secondary schools in the region)

|  | **2018** | **2020** | **2021** | **2022** | **2023** |
| --- | --- | --- | --- | --- | --- |
| **Dublin North** | — | — | 70% (14/20) | — | 85% (17/20) |
| **Cavan** | — | — | 100% (11/11) | — | 100% (11/11) |
| **Monaghan** | — | — | 100% (12/12) | — | 100% (12/12) |
| **Galway city** | 100% (10/10) | 100% (10/10) | — | 100% (10/10) | — |
| **Galway County** | 100% (36/36) | 100% (36/36) |  | 100% (36/36) |  |
| **Mayo** | 100% (26/26) | 100% (26/26) | — | 100% (26/26) | — |
| **Roscommon** | 100% (9/9) | 100% (9/9) | — | 100% (9/9) | — |

**Table S4.** Approximate estimates of the proportion of all eligible secondary school students who responded to the survey.

| **Region** | **Year of survey (Oct-Dec)** | **Number of pupils from 4th and 5th year surveyed** | **Number of junior certificate students registered in participating schools the previous academic year*** | **Proportion surveyed Vs eligible** |
| --- | --- | --- | --- | --- |
| **Galway city** | 2018 | 766 | N/A | N/A |
| **Galway city** | 2020 | 747 | N/A | N/A |
| **Galway city** | 2022 | 854 | 1099 | 78% |
| **Galway County** | 2018 | 1966 | N/A | N/A |
| **Galway County** | 2020 | 1919 | N/A | N/A |
| **Galway County** | 2022 | 2039 | 2699 | 76% |
| **Mayo** | 2018 | 1472 | N/A | N/A |
| **Mayo** | 2020 | 1438 | N/A | N/A |
| **Mayo** | 2022 | 1438 | 1861 | 77% |
| **Roscommon** | 2018 | 515 | N/A | N/A |
| **Roscommon** | 2020 | 623 | N/A | N/A |
| **Roscommon** | 2022 | 677 | 749 | 90% |
| **Cavan** | 2021 | 819 | 1005 | 81% |
| **Cavan** | 2023 | 801 | 1197 | 67% |
| **Monaghan** | 2021 | 834 | 939 | 89% |
| **Monaghan** | 2023 | 895 | 1039 | 86% |
| **Dublin North**** | 2021 | 1457 | 1682 | 87% |
| **Dublin North**** | 2023 | 1666 | 2546 | 65% |
|  |  |  |  | **Mean** = 80%  **SD** = 6% |

* Extracted from the Department of Education records (not available prior to 2021) (<https://www.gov.ie/en/collection/post-primary-schools/>).

**Dublin North numbers are limited to schools that took part in the study. For all other areas, all secondary schools participated.

**Table S5.** Descriptive statistics for the secondary school sample, per survey year

|  | **Ireland West** | | | **Ireland North-East** | |
| --- | --- | --- | --- | --- | --- |
|  | **2018** (N=4722) | **2020** (N=4828) | **2022** (N=5122) | **2021** (N=3160) | **2023** (N=3604) |
| **Local authority** |  |  |  |  |  |
| North Dublin | 0 (0%) | 0 (0%) | 0 (0%) | 1510 (47.8%) | 1858 (51.6%) |
| Cavan | 0 (0%) | 0 (0%) | 0 (0%) | 834 (26.4%) | 822 (22.8%) |
| Monaghan | 0 (0%) | 0 (0%) | 0 (0%) | 816 (25.8%) | 924 (25.6%) |
| Mayo | 1463 (31.0%) | 1479 (30.6%) | 1478 (28.9%) | 0 (0%) | 0 (0%) |
| Roscommon | 511 (10.8%) | 629 (13.0%) | 672 (13.1%) | 0 (0%) | 0 (0%) |
| Galway County | 1975 (41.8%) | 1966 (40.7%) | 2097 (40.9%) | 0 (0%) | 0 (0%) |
| Galway City | 773 (16.4%) | 754 (15.6%) | 875 (17.1%) | 0 (0%) | 0 (0%) |
| **Gender** |  |  |  |  |  |
| Male | 2337 (49.5%) | 2360 (48.9%) | 2505 (48.9%) | 1633 (51.7%) | 1788 (49.6%) |
| Female | 2358 (49.9%) | 2374 (49.2%) | 2469 (48.2%) | 1377 (43.6%) | 1665 (46.2%) |
| Trans/Gender-diverse | 0  (0%)* | 77  (1.6%) | 139  (2.7%) | 130 (4.1%) | 134 (3.7%) |
| Missing | 27  (0.6%) | 17  (0.4%) | 9  (0.2%) | 20  (0.6%) | 17  (0.5%) |
| **Age group** |  |  |  |  |  |
| 15 years | 1439 (30.5%) | 1341 (27.8%) | 1452 (28.3%) | 1014 (32.1%) | 1533 (42.5%) |
| 16 years | 3025 (64.1%) | 3094 (64.1%) | 3109 (60.7%) | 1595 (50.5%) | 1796 (49.8%) |
| 17-19 years | 240  (5.1%) | 378 (7.8%) | 560 (10.9%) | 531 (16.8%) | 252 (7.0%) |
| Missing | 18  (0.4%) | 15  (0.3%) | 1  (0.0%) | 20  (0.6%) | 23  (0.6%) |
| **Family make-up** |  |  |  |  |  |
| Two Parents | 3749 (79.4%) | 3845 (79.6%) | 3973 (77.6%) | 2438 (77.2%) | 2779 (77.1%) |
| One Parent | 871 (18.4%) | 834 (17.3%) | 825 (16.1%) | 614 (19.4%) | 600 (16.6%) |
| Other | 84  (1.8%) | 126 (2.6%) | 312  (6.1%) | 85  (2.7%) | 204 (5.7%) |
| Missing | 18  (0.4%) | 23  (0.5%) | 12  (0.2%) | 23  (0.7%) | 21  (0.6%) |
| **Maternal education** |  |  |  |  |  |
| Third level degree | 2415 (51.1%) | 2482 (51.4%) | 3380 (66.0%) | 1953 (61.8%) | 2398 (66.5%) |
| Secondary School | 1188 (25.2%) | 1080 (22.4%) | 1233 (24.1%) | 767 (24.3%) | 700 (19.4%) |
| Primary School | 337  (7.1%) | 261 (5.4%) | 94  (1.8%) | 49  (1.6%) | 61  (1.7%) |
| Not known | 751 (15.9%) | 949 (19.7%) | 402  (7.8%) | 369 (11.7%) | 420 (11.7%) |
| Missing | 31 (0.7%) | 56 (1.2%) | 13 (0.3%) | 22 (0.7%) | 25 (0.7%) |

*No transgender/gender diverse options provided in 2018

## Supplementary Figures

**Figure S1.** Map of participating regions in Planet Youth Ireland up to 2023.

**Figure S2.** Comparison of sample demographics to national census (2022) statistics for children aged 0-18


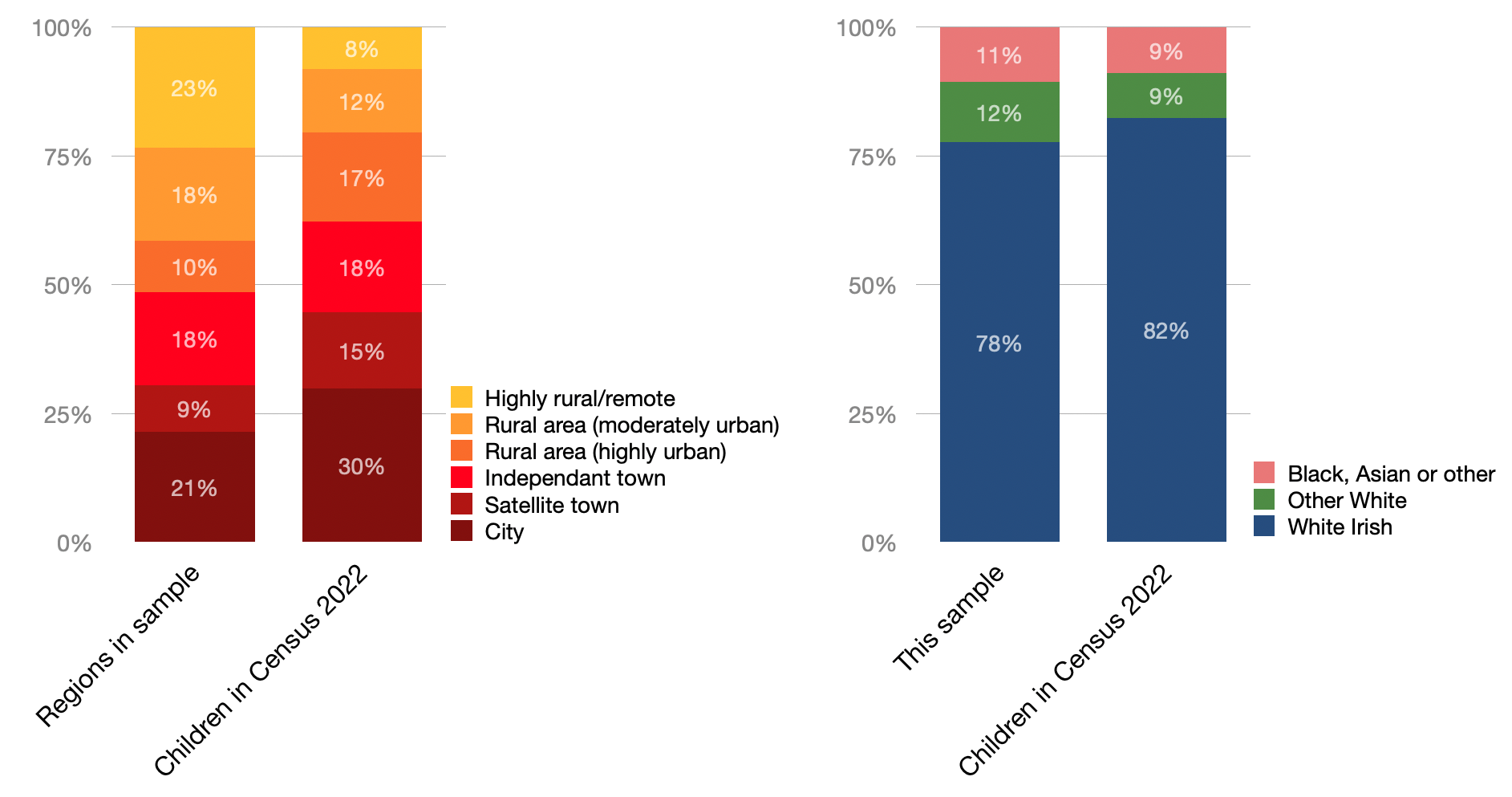


**Figure S3**. National prevalence estimates for each mental health outcome, with variation by school (grey circles) and region (black circles with error bars). Sample sizes in each analysis: 20,920; 20,481; 20,438.


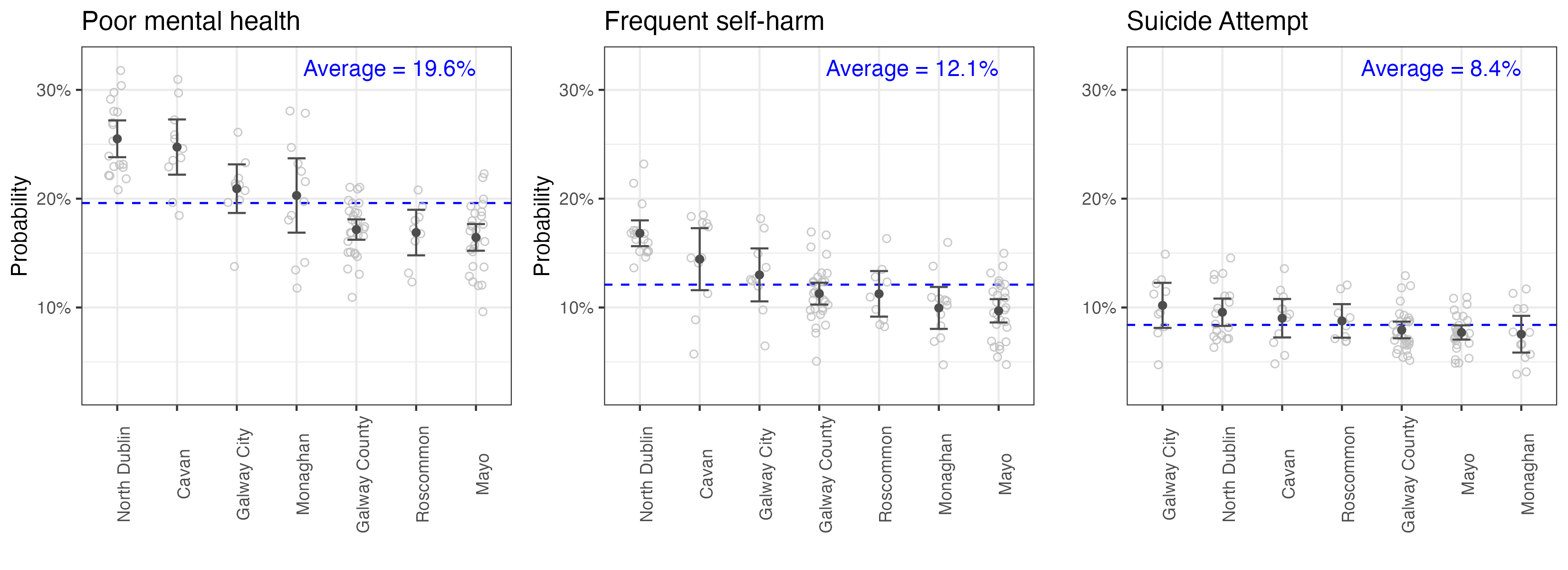


**Figure S4**. Unadjusted prevalence of each outcome, split by gender, for the COVID-19 period (2020-2021) and 2 years later (2022-2023)**
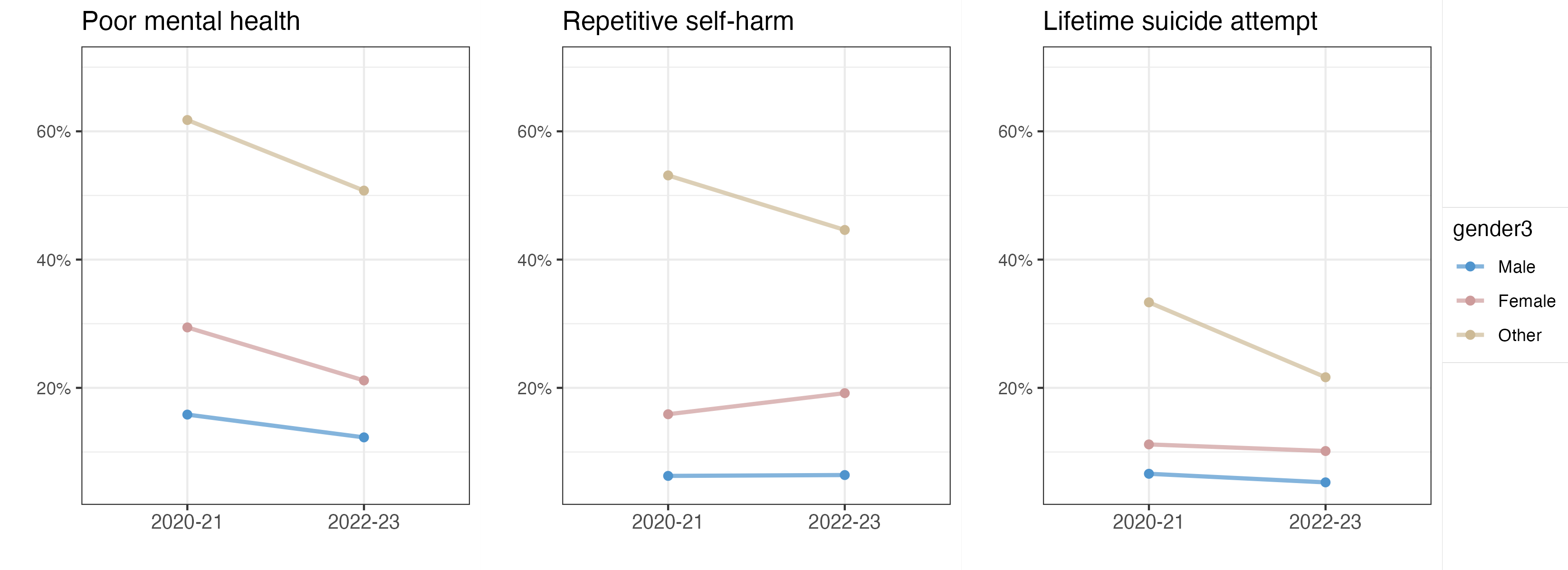
**

**Figure S5.** Trends of psychological distress in secondary students for all genders (top) and male and females only (bottom)


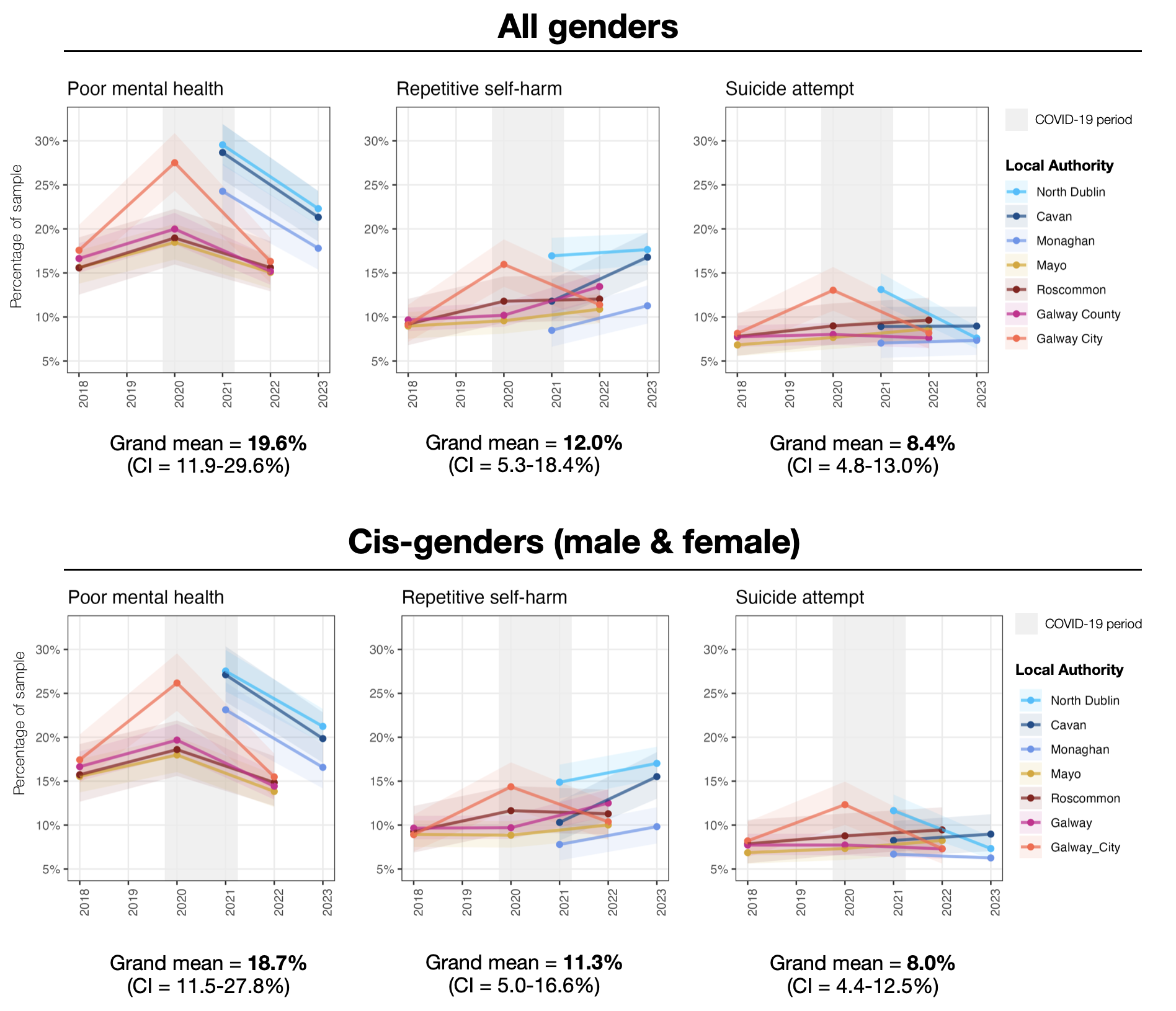


**Supplementary results**

The proportion of trans/gender-diverse individuals in the sample changed over time, and they had particularly high rates of all outcomes across the 5-year study period (Fig 2; Table S5)., We re-ran analyses without this group to ensure trends were not an artefact of changing demographics (new N = 20,866). As in the primary results including all genders, the proportion of adolescents reporting poor mental health decreased significantly from 2020/21 to 2022/23 (OR = 0.68, 95% CI = 0.63-0.74, p < .001), as did the proportion reporting lifetime suicide attempt (OR = 0.87, 95% CI = 0.77 – 0.98, p = 0.02). The significant rise in repetitive self-harm was also observed in this restricted sample (OR = 1.19, 95% CI = 1.08 – 1.32). Also consistent with primary results, rates of self-harm did not return to their pre-pandemic levels but showed significant increase from 2018 to 2022 in regions with data across these years (OR = 1.28, 95% CI = 1.11-1.48). In the intercept-only analysis, which was used to estimate average prevalence of each psychological distress measure, intraclass correlation coefficients (ICCs) representing clustering within schools and local authorities did not differ from primary results, when corrected to 0.01 decimal points (all ICCs < 5%).
